# Access to food outlets and children's nutritional intake in urban China: a difference-in-difference analysis

**DOI:** 10.1186/1824-7288-38-30

**Published:** 2012-06-30

**Authors:** Rui Wang, Lu Shi

**Affiliations:** 1 UCLA Luskin School of Public Affairs, 3250 Public Affairs Building, Box 951656, Los Angeles, CA, 90095, USA; 2 UCLA School of Public Health, 61-253 CHS, 650 Charles E. Young Drive S, Los Angeles, CA, 90095, USA

**Keywords:** Food environment, Nutrition, Child, Chinese city

## Abstract

**Background:**

In recent years supermarkets and fast food restaurants have been replacing those “wet markets” of independent vendors as the major food sources in urban China. Yet how these food outlets relate to children’s nutritional intake remains largely unexplored.

**Method:**

Using a longitudinal survey of households and communities in China, this study examines the effect of the urban built food environment (density of wet markets, density of supermarkets, and density of fast food restaurants) on children’s nutritional intake (daily caloric intake, daily carbohydrate intake, daily protein intake, and daily fat intake). Children aged 6–18 (n = 185) living in cities were followed from 2004 to 2006, and difference-in-difference models are used to address the potential issue of omitted variable bias.

**Results:**

Results suggest that the density of wet markets, rather than that of supermarkets, positively predicts children’s four dimensions of nutritional intake. In the caloric intake model and the fat intake model, the positive effect of neighborhood wet market density on children’s nutritional intake is stronger with children from households of lower income.

**Conclusion:**

With their cheaper prices and/or fresher food supply, wet markets are likely to contribute a substantial amount of nutritional intake for children living nearby, especially those in households with lower socioeconomic status. For health officials and urban planners, this study signals a sign of warning as wet markets are disappearing from urban China’s food environment.

## Background

China is now experiencing the world’s fastest growth in supermarkets (e.g. Carrefour, Wal-Mart and their local clones), with sales at these stores growing by as much as 40 percent annually [1]. Asia’s traditional “wet markets” (also called “open markets” or “free markets”), where independent small vendors in separate stalls sell live animals and fresh produce to customers, increasingly face competition from supermarkets that supply more manufactured foods with more salt and sugar added [2]. In many Asian countries, the traditional food retail channel of wet markets has shown considerable resilience in its competition against supermarket chains [3-7]. Yet in the case of the mainland China, local governments seem to favor supermarkets over wet markets [8]. Aside from possible incentives in local fiscal revenue, replacing wet markets with supermarkets might be justified by the stated goal of increasing hygiene standards, as the former was often identified as an important source of emerging infectious diseases [9]. Another changing aspect of China’s food environment is the expansion of fast food restaurants supplying Western and Chinese variants of pizza, hamburgers, fried chicken, etc., that tend to provide food and drinks with higher fat and sugar content [10]. The expansion of fast food restaurants could have been helped by increased income, lower opportunity cost of time and lower cost of transporting goods [11]. These Western fast food restaurants are quickly followed by local food chains that imitate their models [12,13].

The relationship between the food environment and health behaviors and/or outcomes (mainly fast food consumption, overweight, etc.) has been documented in recent literature [14-18]. However, for a better understanding of what the above changes in food environment could mean for a developing country, more empirical evidence is needed for at least two reasons. First, the majority of current evidence is based on cross-sectional data and only confirms the correlations between land use patterns (e.g., density, mixture of different land use types, and design of street networks) and health, which might be biased by residential self-selection – people self-select into the community they choose to live in [19]. Second, almost all of the above-mentioned empirical studies are based upon data from industrialized countries, where eating behavior, health background and the speed of land use change are often very different from those of developing countries. For instance, undesirable food environment in the developed nations are usually linked with overweight among children and adults, yet in many developing nations protein-energy malnutrition remain the dominant public health threat [20]. In addition, cities in emerging economies such as China have been rapidly growing in the recent past. The spatial and socio-economic structure of Chinese cities has been dramatically changed with on average more than 10 million new urban dwellers added annually during the past two decades.

Interest in China’s built environment and population health has been developing among academics. For instance, Van de Poel et al. [21] find that, in China, an aggregated measure of urbanization are associated with average risks of overweight and hypertension, controlling for individual demographic and socio-economic status. However, for a better understanding of the causal pathways from food access to health outcomes in the context of developing countries, we need to address multiple disaggregated components, including the research gap on food environment and nutritional intake. In this study, we use a longitudinal dataset to examine the effect of easy access to fast food restaurants, supermarkets and wet markets on children’s nutritional intake in Chinese cities. We are interested in whether living in a neighborhood with higher density of food outlets is associated with more carbonhydrate, fat, protein and total caloric intake among children. Given the popular local policy of replacing wet markets with supermarkets, we are also interested in testing whether the effect of wet market density differs significantly from that of supermarket density.

## Methods

### Data

This study uses the China Health and Nutrition Survey (CHNS), a multi-wave ongoing longitudinal survey conducted jointly by the Carolina Population Center and China’s Ministry of Health. The CHNS dataset available to us consists of seven waves (1989, 1991, 1993, 1997, 2000, 2004, and 2006). It employs a multistage random cluster sampling process to draw households from urban (and rural) areas in nine geographically and economically different provinces: Guangxi, Guizhou, Heilongjiang, Henan, Hubei, Hunan, Jiangsu, Liaoning, and Shandong. This dataset provides detailed socio-economic, nutrition, and health information of households and individuals. In addition, it includes separate community questionnaires collecting information (e.g., accessibility) on community built environment or resources, such as transit, recreation facility, public space, school/nursery, hospital, supermarket, wet market, fast food restaurant, etc., at the neighborhood (i.e., the administrative unit of residential committees, typically called “juweihui” in Chinese) level. Most built environment variables are only available for the last three waves. A comprehensive review of the CHNS dataset can be found in the description by Popkin et al. [22].

The urban subsample of CHNS includes communities from urban districts of prefecture-level cities and towns that are county seats. A total of 9,543 individuals from 2,473 households and 86 neighborhoods are identified in this multi-wave subsample. In the urban subsample, slightly less than half of the observations (46% of individuals and 49% of neighborhoods) are from prefecture-level cities, with the rest from county-level cities. The neighborhoods vary in size of their areas, with a median of 1.1 km^2^, a 25 percentile of 0.5 km^2^, and a 75 percentile of 3 km^2^. The population sizes of neighborhoods also vary, with a median of 2,400, a 25 percentile of 1,470, and a 75 percentile of 3,787.

This study uses longitudinal data of school-age children (between 6 and 18)^a^ and their households and neighborhoods from the 2004 and 2006 waves, when neighborhood food environment data are available. To rule out likely outlier or potentially misidentified urban neighborhoods, analyses are restricted to neighborhoods within 100 minutes of bicycling distance from nearest major medical facility (>99% of the full urban household sample), and within 25 km from a park (96% of the full urban household sample).^b^ The overall attrition rate of urban individuals is 17% from 2004 to 2006. Among 997 observed urban children aged between 6 and 18 in the waves of 2004 and 2006, there are 373 aged between 6 and 18 in 2004, among which 303 were followed in 2006, indicating an attrition rate of about 19%. However, the dataset does suffer some loss from missing values. After we dropped observations who ever answered “don’t know” or “refused to answer” in our variable list, the longitudinal model is left with 185 children. Food consumption data have been collected for three consecutive days, and individuals’ food consumption information were then used to calculate the nutrient values (total caloric intake, total protein intake, total fat intake and total carbohydrate intake) based on the 2004 Food Composition Table for China [23].

Compared to data used in existing literature, the significant potential and advantage of the CHNS data can be summarized in two aspects. First, it offers longitudinal data, providing a chance for identifying causal effects. As previously mentioned, very few of the existing studies on the relationship between land use/built environment and behavior/health use randomized longitudinal datasets. Second, the data offers high quality income information on each individual, representing a significant advance in the measurement of income in China [24]. Questions on income and time allocation probe for any possible activity each person might have engaged in during the previous year, both in and out of the formal market. Information on state-subsidized housing is gathered from respondents to generate in-kind income, so that full income from market and non-market activities is imputed, and is adjusted by provincial consumer price indexes.

### Model and key variables

The focus of this study is to quantify how neighborhood environment affects nutritional intake of Chinese urban children aged 6–18. The independent effect of a change in neighborhood environment (treatment, T) on individual i is defined by Y_i,t0,T_ − Y_i,t0_, which is counterfactual and unobservable. The difference-in-differences (DID) method is employed to estimate individual average treatment effects using the two-wave longitudinal data from CNHS.

The DID model can be summarized as ΔY_it_ = ΔX_it_β + ΔZ_it_γ + Δu_it_, with t = 1, 2, where u_it_ is a time-varying household/individual error. Y_it_ includes the nutritional intake variables of the child i at time t. X_it_ is a vector of observable individual/household characteristics. Z_it_ is a vector of food environment variables of the household’s neighborhood, including three variables indicating the neighborhood densities of supermarkets, wet markets, and fast food restaurants, as obtained from the questionnaire items in the CHNS community survey (“how many supermarkets/wet markets/fast food restaurants are there in your neighborhood?”). As access to vehicular transportation has been shown to be linked with access to vegetable and fruits [25], we include two transportation-related variables in our covariates (X_it_) list: whether the neighborhood has any bus stop and whether the household owns a vehicle. In addition, since the choice of wet markets as a shopping destination is linked with affordability [7,26], we interact the household income variable with the neighborhood wet market density to see whether household income affects the association between wet market access and the child’s nutritional intake. The other interaction term in the models is the interaction between being under the age of 12 and the fast food restaurant density, to account for the possible difference in fast food consumption between younger children and adolescents.

As a comparison, ordinary least squares (OLS) regressions using the 2004 and 2006 cross-sectional samples are conducted to illustrate how different between focusing on the longitudinal dimension (e.g., the within differences) of the data and running cross-sectional regressions. We add maternal education (in years) and the child’s gender to the OLS models, as these are important predictors of the child’s health behavior that are unlikely to vary between 2004 and 2006 (i.e., the individual fixed effects in DID models account for such time-invariant variables). This helps us identify whether unobserved heterogeneity exists and affects the results. Considering the relatively limited attrition (due to moving, death, etc.) rate, selection bias is assumed to be a minor concern in this analysis.

To better understand the possible difference between supermarkets and wet markets in terms of children’s nutritional intake, we also test the hypothesis that the number of supermarkets within five kilometers has a different effect from that of the number of wet markets within five kilometers. We do not test the difference between the fast food restaurant density and the other two food environment variables since we believe that restaurants belong to a qualitatively different food outlet category, not directly comparable to wet markets and supermarkets.

## Results

Table 1 provides the descriptive statistics of our longitudinal sample and Table 2 describes the OLS and DID model estimates. After controlling for the effects of individual children and their households, the density of wet markets is positively associated with all four different measurements of nutrition intake. In the four DID models, one additional wet market within five square kilometers is associated with an increase of 31.41 kcal in daily caloric intake (p < 0.01), 3.38 grams of carbohydrate in daily carbohydrate intake (p < 0.1), 1.339 grams of protein in daily protein intake (p < 0.01), and 1.412 grams of fat in daily fat intake (p < 0.01).

**Table 1:** Descriptive statistics of the study sample

**Variable**	**Definition**	**2004**		**2006**	
		**Mean**	**Std. Dev.**	**Mean**	**Std. Dev.**
*Outcome variables*					
Kilocalorie	Average daily calorie intake (in kilocalories)	1,909.53	608.63	1,958.93	627.33
Carbohydrate	Average daily carbohydrate intake (in grams)	255.70	95.81	268.68	92.46
Protein	Average daily protein intake (in grams)	62.80	24.74	63.87	23.45
Fat	Average daily fat intake (in grams)	70.52	32.33	70.31	36.01
*Independent variables*					
Age	Calculated age in years	11.43	2.73	13.40	2.72
Household income per capita	Per capita household income (in thousand RMB at 2006)	6.55	6.01	7.13	6.68
Supermarket density	Number of supermarkets within 5 km of neighborhood	6.59	5.87	3.08	3.25
Wet market density	Number of wet markets within 5 km of neighborhood	6.25	7.88	4.17	4.57
Fast food restaurant density	Number of fast food restaurants (e.g. McDonald’s, KFC) in neighborhood	1.02	2.68	0.45	2.35
Owning car	Household automobile ownership (own = 1, not own = 0)	0.04	0.19	0.07	0.26
Bus stop	Existence of bus stop(s) in neighborhood (yes = 1, no = 0)	0.70	0.46	0.77	0.42
Female	Female = 1, male = 0	0.51	0.50	0.51	0.50
Maternal education	Mother’s education in years	10.84	2.87	10.84	2.87
Child dummy	1 if aged 6–12, 0 if aged 13–17	0.66	0.48	0.45	0.50

**Table 2:** Average daily caloric, carbohydrate, protein, and fat intakes of children

	**Kilocalorie**			**Carbohydrate**			**Protein**			**Fat**		
	**OLS ‘04**	**OLS ‘06**	**DID**	**OLS ‘04**	**OLS ‘06**	**DID**	**OLS ‘04**	**OLS ‘06**	**DID**	**OLS ‘04**	**OLS ‘06**	**DID**
Age	35.09 (.798)	467.60*** (.008)	1275.00* (.069)	18.19 (.325)	77.33*** (.001)	157.80 (.159)	-3.23 (.587)	17.37*** (.010)	53.30** (.047)	-2.77 (.736)	10.15 (.357)	45.89 (.231)
Age squared	1.88 (.760)	-16.04** (.014)	-10.67** (.038)	-.27 (.748)	-2.67*** (.003)	-1.72** (.037)	.24 (.373)	-.61** (.014)	-.22 (.27)	.22 (.532)	-.34 (.417)	-.33 (.243)
Income per capita	35.58*** (.000)	7.45 (.436)	15.02 (.107)	4.28** (.012)	.56 (.688)	1.28 (.388)	1.22** (.014)	.38 (.416)	.39 (.276)	1.51* (.055)	.40 (.504)	.94* (.067)
Wet market density	44.39*** (.001)	4.75 (.760)	32.25*** (.005)	6.80*** (.006)	-.53 (.825)	3.43* (.057)	1.60*** (.008)	-.07 (.909)	1.35*** (.002)	1.20* (.089)	.79 (.337)	1.47** (.017)
Income * wet market density	-4.00*** (.004)	.33 (.904)	-2.72** (.044)	-.60** (.018)	-.05 (.900)	-.27 (.213)	-.10 (.129)	.10 (.364)	-.07 (.150)	-.13* (.079)	.018 (.920)	-.15** (.041)
Supermarket density	-.83 (.906)	12.81 (.414)	-6.26 (.480)	-.06 (.967)	1.86 (.471)	.061 (.965)	-.11 (.672)	.13 (.808)	-.38 (.260)	-.013 (.974)	.54 (.584)	-.61 (.208)
Fast food restaurant density	.23 (.990)	-31.49* (.070)	-9.63 (.737)	-.37 (.880)	-2.67 (.543)	-.49 (.914)	-.85 (.265)	-.29 (.463)	.01 (.996)	.57 (.698)	-2.19*** (.000)	-.82 (.601)
Interaction of being under 12 and fast food restaurant density	-32.21 (.182)	15.89 (.505)	20.85 (.617)	-4.80* (.093)	0.67 (.890)	-1.02 (.878)	-.32 (.729)	.79 (.399)	.46 (.772)	-1.31 (.481)	1.08 (.258)	2.57 (.260)
Owning car	-53.74 (.659)	78.97 (.512)	208.90 (.292)	-30.19 (.293)	20.49 (.353)	37.03 (.242)	1.64 (.807)	6.07 (.190)	10.04 (.185)	6.81 (.611)	-2.74 (.711)	2.59 (.811)
Bus stop	-84.03 (.289)	65.59 (.539)	147.80 (.206)	3.32 (.797)	13.16 (.399)	32.46* (.082)	-3.84 (.274)	-1.62 (.713)	7.22 (.106)	-9.08* (.066)	2.14 (.721)	-1.32 (.836)
Maternal education	-21.36* (.086)	14.19 (.487)		-4.98** (.031)	-.94 (.723)		.23 (.663)	.53 (.489)		-.26 (.826)	1.79 (.132)	
Female	-274.60*** (.001)	-363.70*** (.000)		-40.26*** (.001)	-50.36*** (.000)		-14.52*** (.000)	-9.94*** (.005)		-6.18 (.240)	-13.56** (.014)	
Adj./within R2	.36	.18	.11	.35	.18	.09	.31	.16	.13	.12	.10	.07
Prob. > F	.00	.00	.04	.00	.00	.09	.00	.00	.01	.01	.00	.36

Note: N = 185 for OLS regressions; N = 370 for DID regressions; *p < .10, **p < .05, ***p < .01; p-values included in parentheses; robust standard errors are used in OLS regressions.

As for the interaction term of neighborhood wet market density and the household income per capita, our DID models show that every 1,000 Yuan increase in household income per capita reduces the neighborhood wet market’s effect on caloric intake (by 2.74 kcal per day, p < 0.1) and on fat intake (by 0.15 gram of fat per day, p < .05), indicating accessibility to wet markets has larger positive caloric/fat intake effect on children from households of lower income. Similar effects are observed for the interaction terms in our protein intake model and carbohydrate intake model, although the effects are not statistically significant there.

On the other hand, even though the number of fast food restaurants within five kilometers of residence is significantly associated with nutritional intake in all four OLS models, neither the number of fast food restaurants nor the number of supermarkets significant predict nutritional intake in any of the four DID models. This may indicate that the association between fast food restaurants and nutritional intake in the OLS models could be due to omitted variable biases.

F-tests conducted after the DID models show that the effect of supermarket density is significantly different from that of wet market density in our total caloric intake model (F = 5.05, p = 0.025), protein intake model (F = 7.87, p = 0.005), and fat intake model (F = 5.23, p = 0.023), but not in carbohydrate model (F = 1.72, p = 0.19).

As for the transportation covariates, having bus stops in the neighborhood is positively associated with carbohydrate intake (p < 0.05) and protein intake (p < 0.05), whereas the household’s car ownership does not significantly predict any of our dependent variables in Table 2.

In our OLS models, being female significantly predicts less nutritional intake, for all four measures in 2004 and 2006, with the only exception that in 2004 this effect is statistically insignificant for the OLS fat intake model (p = 0.24). In 2004 more maternal education is associated with less total caloric intake with a p value of 0.084 and less carbonhydrate intake with a p value of 0.031, but this pattern is not seen in the other two 2004 OLS models, nor is it seen in any of the four 2006 OLS models.

## Discussion

Unlike most of the earlier studies, our DID model addresses the “residential self-selection bias” and demonstrated an independent association between neighborhood food environment and specific nutritional intake among children. The government-led conversion of wet markets into supermarkets has been carried out in Chinese cities since the early 2000s since these wet markets are often considered as unclean for urban environment and inefficient in generating circulation tax revenue. As our study shows, however, the density of wet markets is the only one of the three food environment variables that positively predict protein intake, which serves a vital nutrient for the younger population’s physical development and even cognitive function [27]. For health officials and urban planners, this should signal a sign of warning as wet markets are disappearing from Urban China’s food environment (in many cases as a result of local governments’ attempt to replace them with supermarkets). When new food outlets like fast food restaurants have been shown to be linked with undesirable health outcomes in the population [28], it might be a risky approach to displace the traditional affordable food sources like wet markets. As for the hygiene risks associated with the live poultry in wet markets, measures like separating animals in different stalls and booths, discouraging the transaction of wild-caught animals and the first use of pandemic vaccines in farms that supply animals to wet markets can be expected to reduce the infectious disease risk. Meanwhile, the significant association we found between wet market density and the other three nutritional intake measures (fat, total calories and carbonhydrate), at a time when China’s childhood obesity increases fast, implies that parents and teachers might want to know better what food children are getting from wet markets.

Some results from our difference-in-difference model also help explain the documented association between living in households of higher socio- economic status and higher body-mass-index among Chinese children: for instance, higher household income per capita significantly predicts more fat intake in our DID fat model. Having a bus stop in the neighborhood is also associated with more nutrient intake of carbonhydrate, and having a bus nearby is often a measure of the community’s degree of urbanization. Thus, we might infer that the significant coefficient of “bus stop in the neighborhood” in our carbonhydrate DID model could reflect the extra carbonhydrate intake among children who live in more urbanized and developed parts of Urban China, another factor that could have contributed to the higher BMI among richer children.

On the other hand, as we observe that the interaction term between household income per capita and wet market density is negatively associated with caloric intake and fat intake, we should also beware that accessibility to wet market foods could have a stronger effect on the caloric/fat intake among children from lower-income families, perhaps from their relatively lower prices. This is consistent with previous literature that income effect on potentially detrimental diet is stronger with poor households in China [29]. Thus, even though China is still at the stage when high household income positively predicts high caloric/fat intake (as confirmed by our DID models) and high body mass index [30], certain segments of low-income population could also be at risk for excessive caloric/fat intake as well, because their children’s caloric/fat intake may be more sensitive to income growth.

There are, of course, limitations in this study. Local food environment is measured by the relatively loose spatial unit of “neighborhood” instead of strict area density. Limited waves of built-environment measures and missing variable values constrain the sample size. Possibly self-selected attrition of the sampled children could also have biased the results to some extent. In addition, we measured the density of food outlets by the number of outlets in the community without paying attention to the actual size of each type of food outlets, which might have missed some important information about the child’s actual access to foods. For instance, we notice from Table 1 that the number of supermarkets in the neighborhood declined from 2004 to 2006 at a time when overall supermarkets’ market share increased among all food outlets, which might be a result of China’s ongoing trend of supermarket consolidation [8] but we do not know whether the actual supermarket size increased since 2004. Similarly, we do not know what contributed to the observed decline of fast food restaurant density as revealed by Table 1, though we suspect that a growth in the average revenue per restaurant coupled with industry-wide consolidation could explain this decline during the fast food industry’s boom. Future studies would benefit from a larger size of longitudinal sample, better measures of the food environment, and a more complete control of covariates including food price. In addition, studies like this one should be combined with studies on the relationship between nutritional intake and health outcomes to help us better understand the welfare implications of policies on food environment.

## Endnotes

^a^Children of school age and those younger may have substantially different daily activity, nutritional demand, and food intake pattern. We select the age range between 6 and 18 so that the behavioral pattern of children in our sample is relatively homogeneous.

^b^We did so by examining the data distribution. A very small number of observations are located in places far away from major medical facility or a park. We consider them as not located in typical urban environment

## Competing interests

The authors certify that there is no conflict of interest to the best of their knowledge.

## Authors’ contributions

RW started the study and designed the analysis framework. LS conceptualized the research questions and hypotheses. Both authors read and approved the final manuscript.

## References

[B1] Hu D Reardon T Rozelle S Timmer P Wang H The emergence of supermarkets with Chinese characteristics: challenges and opportunities for China's agricultural development Dev Policy Rev 2004 22 557 86 10.1111/j.1467-7679.2004.00265.x

[B2] Reardon T Timmer P Berdegue J The rapid rise of supermarkets in developing countries: induced organizational, institutional, and technological change in agrifood systems J Agric Dev Econ 2004 1 168 83

[B3] Goldman A Supermarkets in China: entry limitations and strategic dilemmas MKTG 96.080, China Europe International Business School 1996 96.080 1 3

[B4] Goldman A Krider R Ramaswami S The persistent competitive advantage of traditional food retailers in Asia: wet markets’ continued dominance in Hong Kong J Macromark 1999 19 2 126 139 10.1177/0276146799192004

[B5] Goldman A Supermarkets in China: the case of Shanghai Int Rev Retail Distrib Consum Res 2000 10 1 1 21 10.1080/095939600342370

[B6] Goldman A Ramaswami S Krider RE Barriers to the advancement of modern food retail formats: theory and measurement J Retail 2002 78 4 281 295 10.1016/S0022-4359(02)00098-2

[B7] Zhang X The dynamics of Chinese consumers Journal of International Food & Agribusiness Marketing 2003 14 47 66 10.1300/J047v14n01_04

[B8] He J A comparison of “converting free markets into supermarkets” across large and middle-sized cities (大中城市“农改超”模式比较) Business Time 2006 31 11 12

[B9] Yu H Feng Z Zhang X Xiang N Huai Y Zhou L Li Z Xu C Luo H He J Guan X Yuan Z Li Y Xu L Hong R Liu X Zhou X Yin W Zhang S Shu Y Wang M Wang Y Lee C-K Uyeki TM Yang W Human influenza A (H5N1) cases, urban areas of People’s Republic of China, 2005–2006 Emerg Infect Dis 2007 13 7 1061 1064 10.3201/eid1307.061557 18214180 PMC2878233

[B10] Bowman SA Gortmaker SL Ebbeling CB Pereira MA Ludwig DS Effects of fast-food consumption on energy intake and diet quality among children in a National Household Survey Pediatrics 2004 113 112 118 10.1542/peds.113.1.112 14702458

[B11] Reardon T Timmer CP Barrett CB Berdegué J The rise of supermarkets in Africa, Asia, and Latin America Am J Agric Econ 2003 85 5 1140 1146 10.1111/j.0092-5853.2003.00520.x

[B12] Wang Y Monteiro C Popkin BM Trends of obesity and underweight in older children and adolescents in the United States, Brazil, China, and Russia Am J Clin Nutr 2002 75 6 971 977 12036801 10.1093/ajcn/75.6.971

[B13] Lobstein T Baur L Uauy R Obesity in children and young people: a crisis in public health Obes Rev 2004 5 Suppl 1 4 104 15096099 10.1111/j.1467-789X.2004.00133.x

[B14] Eid J Overman HG Puga D Fat city: questioning the relationship between urban sprawl and obesity J Urban Econ 2008 63 385 404 10.1016/j.jue.2007.12.002

[B15] Jeffery R Baxter J McGuire M Linde J Are fast food restaurants an environmental risk factor for obesity? Int J Behav Nutr Phys Act 2003 3 1 2 16436207 10.1186/1479-5868-3-2 PMC1397859

[B16] Papas MA Alberg AJ Ewing R Helzlsouer KJ Gary TL Klassen AC The built environment and obesity Epidemiol Rev 2007 29 129 43 10.1093/epirev/mxm009 17533172

[B17] Raja S Yin L Roemmich J Ma C Epstein L Yadav P Ticoalu A Food environment, built environment, and women’s BMI: evidence from Erie County, New York J Plan Educ Res 2010 29 444 460 10.1177/0739456X10367804

[B18] Pearce J Witten K Bartie P Neighbourhoods and health: a GIS approach to measuring community resource accessibility J Epidemiol Community Health 2006 60 389 95 10.1136/jech.2005.043281 16614327 PMC2563982

[B19] Mokhtarian PL Cao X Examining the impacts of residential selection on travel behavior: a focus on methodologies Trans Research Part B 2008 42 204 28 10.1016/j.trb.2007.07.006

[B20] Muller O Krawinkel M Malnutrition and health in developing countries CMAJ 2005 173 279 286 10.1503/cmaj.050342 16076825 PMC1180662

[B21] Van de Poel E O’Donnell O Van Doorslaer E Urbanization and the spread of diseases of affluence in China Econ Hum Biol 2009 7 2 200 216 10.1016/j.ehb.2009.05.004 19560989

[B22] Popkin BM Will China’s nutrition transition overwhelm its health care system and slow economic growth? Heal Aff 2008 27 4 1064 1076 10.1377/hlthaff.27.4.1064 PMC2447919 18607042

[B23] Benjamin D Brandt L Giles J Wang S Income inequality during China’s economic transition China’s great economic transformation 2008 New York: Cambridge University Press

[B24] Popkin BM Du S Zhai F Zhang B Cohort profile: the China Health and Nutrition Survey–monitoring and understanding socio-economic and health change in China, 1989–2011 Int J Epidemiol 2010 39 6 1435 1440 10.1093/ije/dyp322 19887509 PMC2992625

[B25] Sharkey JR Horel S Dean WR Neighborhood deprivation, vehicle ownership, and potential spatial access to a variety of fruits and vegetables in a large rural area in Texas Int J Health Geogr 2010 9 26 10.1186/1476-072X-9-26 20500853 PMC2881903

[B26] Consumer Council Where to shop for food- supermarkets or wet markets? Choice 2002 April 30 34

[B27] Jakobsen LH Kondrup J Zellner M Tetens I Roth E Effect of a high protein meat diet on muscle and cognitive functions: a randomised controlled dietary intervention trial in healthy men Clin Nutr 2011 30 3 303 311 10.1016/j.clnu.2010.12.010 21239090

[B28] Pereira MA Kartashov AI Ebbeling CB Fast-food habits, weight gain, and insulin resistance (the CARDIA study): 15-year prospective analysis Lancet 2005 365 9453 36 42 10.1016/S0140-6736(04)17663-0 15639678

[B29] Du S Mroz TA Zhai F Popkin BM Rapid income growth adversely affects diet quality in China–particularly for the poor! Soc Sci Med 2004 59 7 1505 1515 10.1016/j.socscimed.2004.01.021 15246178

[B30] Xu F Yin X Zhang M Leslie E Ware R Owen N Family average income and body mass index above the healthy weight range among urban and rural residents in regional Mainland China Public Health Nutr 2005 8 1 47 51 10.1079/PHN2005653 15705245

